# Surveillance practices and air-sampling strategies to address healthcare-associated invasive mold infections in Society for Healthcare Epidemiology of America (SHEA) Research Network hospitals—United States, 2020

**DOI:** 10.1017/ice.2021.285

**Published:** 2021-07-16

**Authors:** Jeremy A. W. Gold, Brendan R. Jackson, Janet Glowicz, Kenneth R. Mead, Karlyn D. Beer

**Affiliations:** 1 Mycotic Diseases Branch, Division of Foodborne, Waterborne, and Environmental Diseases, Centers for Disease Control and Prevention, Atlanta, Georgia,; 2 Epidemic Intelligence Service, Centers for Disease Control and Prevention, Atlanta, Georgia,; 3 Division of Healthcare Quality Promotion, Centers for Disease Control and Prevention, Atlanta, Georgia; 4 Division of Field Studies and Engineering, National Institute for Occupational Safety and Health, Centers for Disease Control and Prevention, Cincinnati, Ohio

## Abstract

With this survey, we investigated healthcare-associated invasive mold infection (HA-IMI) surveillance and air sampling practices in US acute-care hospitals. More than half of surveyed facilities performed HA-IMI surveillance and air sampling. HA-IMI surveillance was more commonly performed in academic versus nonacademic facilities. HA-IMI case definitions and sampling strategies varied widely among respondents.

Healthcare-associated invasive mold infections (HA-IMIs), including invasive aspergillosis and mucormycosis, cause devastating morbidity and mortality.^1,2^ Previous HA-IMI clusters have been associated with various mold sources, including construction, water leaks, and air filtration issues.^1,3^ Although considered uncommon, the incidence of HA-IMIs in the United States is unknown. Surveillance for these infections is challenging because of difficulties in ascertaining infection sources and the lack of a standardized case definition. The most widely accepted IMI case definition, developed by the European Organization for Research and Treatment of Cancer and the Mycoses Study Group (EORTC/MSG), is complex and excludes certain types of infections relevant to the healthcare setting, namely, cutaneous and wound infections.^4^ Hospitals have employed environmental air sampling techniques as a tool in HA-IMI cluster investigations and prevention efforts, but the optimal approach to analysis and interpretation of such sampling is unknown.^5^ To gauge current practices regarding hospital HA-IMI surveillance and environmental air sampling, we surveyed members of the Society for Healthcare Epidemiology of America (SHEA) Research Network (SRN).

## Methods

The SRN is a consortium of healthcare facilities collaborating on multicenter healthcare epidemiology research projects. On June 29, 2020, a cross-sectional survey was electronically distributed to eligible facilities, defined as US acute-care hospitals participating in SRN.^6^ In total, 5 e-mail reminders were sent, and the survey was closed on September 3, 2020. The survey was reviewed by SRN Review and Research Committee, formatted on Survey Gizmo, and distributed by e-mail to site primary investigators. We summarized the survey responses regarding HA-IMI surveillance practices, air sampling approaches, and existing collaborations among HA-IMI prevention stakeholders. Using Fisher exact tests for proportions, we compared responses between academic and nonacademic hospitals; we considered *P*-values <.05 statistically significant. This activity was reviewed by the CDC and was conducted consistent with applicable federal law and CDC policy (see eg, 45 CFR part 46, 21 CFR part 56; 42 USC §241(d); 5 USC §552a; 44 USC §3501 et seq).

## Results

Among 71 eligible facilities, 37 (52.1%) completed the survey. Most survey respondents were from academic medical centers (n = 25, 67.6%) and reported the presence of an intensive care unit (n = 35, 94.6%), a hematology-oncology unit (n = 30, 81.1%), and a stem cell transplant program (n = 24, 64.9%) (Supplementary Table 1 online).

Overall, 35 (94.6%) of 37 hospitals performed any surveillance for HA-IMIs, either prospectively (n = 24, 68.6%) or retrospectively (n = 11, 31.4%) during a suspected IMI cluster (Table 1). Academic hospitals (n = 20, 83.3%) were more likely than were nonacademic hospitals (n = 4, 36.4%) to perform prospective monitoring for HA-IMIs (*P* = .02) and to have investigated an IMI cluster during 2018–2019 (n = 12 [50.0%] vs 1 [9.1%]; *P* = .03).

The most commonly used IMI case definition was EORTC/MSG (n = 15, 42.9%); 12 hospitals (34.3%) reported using a custom case definition developed in-house and 8 hospitals (22.9%) did not specify an IMI definition. Among facilities using a custom case definition, notable responses included a case-by-case approach based on clinical features (n = 7) and a definition based on test results (eg, culture, histopathology) (n = 2) regardless of clinical correlation. Regarding determination of whether IMIs were healthcare-associated, facilities mentioned time frames (difference between admission date to illness onset date) ranging from 2 days to >2 weeks. One hospital reported that determination of whether an IMI was hospital-associated might depend on the results of environmental samples or presence of recent construction activity.

Overall, 23 (62.2%) hospitals reported performing any type of air sampling for mold (Table 2). Hospitals most commonly performed air sampling in operating rooms (n = 11, 47.8%) and protective environment rooms (n = 10, 43.5%). Approximately half (n = 11, 47.8%) of hospitals with air sampling reported performing routine, ongoing air sampling at specified time intervals under predetermined conditions using a systematic sampling protocol. Most hospitals (n = 30, 81.1%) reported access to industrial hygienist consultation and the presence of a project risk team (n = 31, 83.8%) to review proposed maintenance, renovation, and construction activities that pose an increased risk of generating or releasing microbial contamination (Supplementary Table 2 online).

## Discussion

In our survey of US SRN acute-care hospitals, most facilities performed prospective HA-IMI surveillance (69%) and most utilized air sampling for mold (62%) as part of HA-IMI prevention or investigation efforts. However, both HA-IMI case definitions and approaches to environmental sampling for mold varied substantially among facilities. The relatively high percentage of participants engaged in HA-IMI surveillance likely reflects that respondents were mostly from academic institutions caring for patient populations at high risk for IMIs (eg, patients with hematologic malignancies or receiving stem cell transplants). This finding is consistent with guidelines from the Infectious Diseases Society of America, which strongly recommend that leukemia and transplant centers surveil for cases of invasive mold infection.^7^

Facilities differed in both how IMIs were defined and how they were determined to be healthcare-associated, mirroring the diversity of case definitions that have been applied in previous HA-IMI cluster investigations.^3^ Ascertaining whether IMI cases are healthcare-associated is difficult because patients who develop IMIs often have complicated medical histories with multiple possible exposures and the incubation periods for some mold infections are not well established.^3^ Furthermore, certain hospitals may lack the laboratory capacity for prompt mold species identification. Despite these challenges, systematic surveillance for HA-IMIs is a necessary step in understanding disease burden, quickly identifying potential clusters, and reducing mortality from these infections; therefore, efforts to develop a feasible HA-IMI surveillance approach, including a standardized HA-IMI case definition, should be prioritized.

Hospitals varied substantially regarding whether and how they performed air sampling for mold, reflecting the controversial role of air sampling in preventing HA-IMIs. During previous HA-IMI clusters, air sampling has been helpful in identifying potential targets for remediation or supporting possible epidemiologic links between a mold sources and affected patients.^3^ For such investigations, air sampling is most useful when performed as an adjunct to a detailed environmental assessment and epidemiologic investigation using a well-designed sampling plan, optimally under the guidance of an industrial hygienist with experience participating in microbiological assessments.^3^ Air sampling may also be useful for monitoring mold counts before, during, and after major construction activities, a practice recommended by guidelines in several countries,^8,9^ but it is not currently recommended by the US Healthcare Infection Control Practices Advisory Committee.^10^ The utility of environmental air sampling may be limited by the lack of established threshold values or regulatory levels for mold in air and the lack of widely accepted industry qualification or practice standards for mold assessors and remediators.^5^ Efforts to identify and close knowledge gaps regarding air sampling strategies for mold are needed; in the interim, consensus guidelines based on expert opinion and existing literature might empower acute care facilities to adopt rational approaches to air sampling for mold.

Our findings are limited by our inability to follow up with respondents and our small sample size. The HA-IMI surveillance and air sampling practices reported by the facilities surveyed might not represent practices of acute care hospitals nationwide. We suspect that facilities with interest or prior experience in HA-IMI prevention were more likely to respond to our survey and that HA-IMI surveillance and air sampling for mold may be less common in other hospitals. Nonetheless, our findings underscore the need to develop generalizable strategies for HA-IMI surveillance and for further data to guide rational approaches to air sampling for mold.

## Supplementary Material

supplementary tables

## Figures and Tables

**Table 1.** Surveillance for Healthcare-associated Invasive Mold Infections at Society for Healthcare Epidemiology of America (SHEA) Research Network Acute Care Hospitals (N = 35)^a^ — United States, 2020

			Hospital Type				
	Total (N = 35)		Academic (n = 24)		Nonacademic (n = 11)		
Characteristic	No.	%	No.	%	No.	%	*P* Value
Hospital monitoring for healthcare-associated invasive mold infections							.02
Prospective	24	68.6	20	83.3	4	36.4	
Retrospective, when a cluster of infections is suspected	11	31.4	4	16.7	7	63.6	
During 2018–2019, the hospital investigated ≥1 cluster of invasive mold infections, whether or not cases were suspected to be healthcare associated	13	37.1	12	50.0	1	9.1	.03
Data sources used to routinely monitor for healthcare-associated mold infections							
Culture	28	80.0	21	87.5	7	63.6	.17
Clinician diagnosis	17	48.6	9	37.5	8	72.7	.08
Galactomannan assay	14	40.0	10	41.7	4	36.4	>.99
Histopathology	13	37.1	11	45.8	2	18.2	.15
(1,3)-β-D-Glucan (Fungitell) assay	12	34.3	10	41.7	2	18.2	.26
Radiographic evidence	9	25.7	7	29.2	2	18.2	.69
Polymerase chain reaction	8	22.9	7	29.2	1	9.1	.39
Diagnostic codes (eg, ICD-10)	1	2.9	1	4.2	0	0.0	>.99
Other	5	14.3	3	12.5	2	18.2	.64
Case definition used to define invasive mold infection							.90
EORTC/MSG invasive fungal infections consensus definition	15	42.9	11	45.8	4	36.4	
Custom definition/other surveillance case definition developed in-house	12	34.3	8	33.3	4	36.4	
No answer provided	8	22.9	5	20.8	3	27.3	
Hospital has a system to identify clusters of healthcare-associated invasive mold infection	25	71.4	18	75.0	7	63.6	.69
Using the institution’s case definition, how many healthcare-associated cases of invasive mold infections did the hospital have during 2018–2019							
0	14	40.0	9	37.5	5	45.5	
1–10	12	34.3	10	41.7	2	18.2	
Do not know	1	2.9	1	4.2	0	0.0	
No response	8	22.9	4	16.7	4	36.4	
Using the institution’s case definition, number of invasive mold infections identified during 2018–2019, including healthcare and non-healthcare-associated cases							
0	4	11.4	2	8.3	2	18.2	
1–10	9	25.7	6	25.0	3	27.3	
11–25	4	11.4	2	8.3	2	18.2	
26–50	3	8.6	3	12.5	0	0.0	
>50	2	5.7	2	8.3	0	0.0	
Unknown	13	37.1	9	37.5	4	36.4	

Note. EORTC/MSG, European Organization for Research and Treatment of Cancer/Invasive Fungal Infections Cooperative Group and the National Institute of Allergy and Infectious Diseases Mycoses Study Group.

a 2 hospitals that did not perform monitoring for healthcare-associated invasive mold infections were excluded from this table.

**Table 2.** Practices Regarding Air Sampling for Mold Among Society for Healthcare Epidemiology of America (SHEA) Research Network Acute-Care Hospitals (n = 23)—United States, 2020

Characteristic	No.	%
**Air-sampling type**		
Air particulate sampling	18	78.3
Culture-based analysis^a^	14	60.9
Direct examination samples^b^	10	43.5
**Areas of the hospital in which air sampling is performed**		
Operating rooms	11	47.8
Protective environment rooms (positive pressure rooms for high-risk immunocompromised patients)	10	43.5
Adult intensive care units	8	34.8
Neonatal intensive care units	6	26.1
Pediatric intensive care units	6	26.1
Outdoor location at air intakes or other likely air entry points	6	26.1
Inpatient rooms (non-ICU)	5	21.7
Pharmacy	5	21.7
Common spaces (eg, hallways, waiting rooms, lobbies)	4	17.4
Burn units	3	13.0
**Air-sampling strategy**		
Sampling performed when there is an event that could disturb a mold source (eg, construction, water leakage, or flooding event)	14	60.9
Routine, ongoing air sampling at specified time intervals under pre-determined conditions, according to a systematic sampling protocol	11	47.8
Random air sampling	3	13.0
Other	5	21.7
Presence of a threshold criteria for mold sampling results at which specific action is taken (eg, closing a patient or procedure room)	9	39.1
Analysis of environmental samples for mold performed by a lab that is accredited through the American Industrial Hygiene Association (AIHA) Environmental Microbiology Laboratory Accreditation Program (EMLAP)		
Yes	10	43.5
No	7	30.4
Respondent did not know	6	26.1

a Culture-based analysis (commonly referred to as “viable mold sampling.” Common samplers for this technique include Andersen N6, SAS Super 180, SKC BioStage and Buck BioAire).

b Direct examination samples are commonly referred to as “nonviable sampling,” “spore trapping,” or “total spore count.” These samples are usually collected using an inertial impactor with air sampling cassettes such as Air-O-Cell, Allergenco-D and Cyclex-D).

## References

[1] Vonberg RP, Gastmeier P. Nosocomial aspergillosis in outbreak settings. J Hosp Infect 2006;63:246–254. 16713019 10.1016/j.jhin.2006.02.014

[2] Novosad SA, Vasquez AM, Nambiar A, Notes from the field: probable mucormycosis among adult solid organ transplant recipients at an acute care hospital—Pennsylvania, 2014–2015. Morbid Mortal Wkly Rept 2016; 65:481–482. 10.15585/mmwr.mm6518a5 27171735

[3] Hartnett KP, Jackson BR, Perkins KM, A guide to investigating suspected outbreaks of mucormycosis in healthcare. J Fungi (Basel) 2019. doi: 10.3390/jof5030069. PMC6787571 31344775

[4] Donnelly JP, Chen SC, Kauffman CA, Revision and update of the consensus definitions of invasive fungal disease from the European Organization for Research and Treatment of Cancer and the Mycoses Study Group Education and Research Consortium. Clin Infect Dis 2020;71:1367–1376. 31802125 10.1093/cid/ciz1008 PMC7486838

[5] Hung L-L, Caufield SM, Miller JD. Recognition, evaluation, and control of indoor mold. American Industrial Hygiene Association website. https://online-ams.aiha.org/amsssa/ecssashop.show_product_detail?p_mode=detail&p_product_serno=20912020. Accessed June 28, 2021.

[6] SRN participating facilities. The Society for Healthcare Epidemiology of America website. http://www.shea-online.org/index.php/practice-resources/research/shea-research-network/srn-participating-institutions. Accessed May 23, 2021.

[7] Patterson TF, Thompson GR III, Denning DW, Practice guidelines for the diagnosis and management of aspergillosis: 2016 update by the Infectious Diseases Society of America. Clin Infect Dis 2016;63:e1–e60. 27365388 10.1093/cid/ciw326 PMC4967602

[8] Talento AF, Fitzgerald M, Redington B, O’Sullivan N, Fenelon L, Rogers TR. Prevention of healthcare-associated invasive aspergillosis during hospital construction/renovation works. J Hosp Infect 2019;103:1–12. 30629998 10.1016/j.jhin.2018.12.020

[9] Risk of fungal infections and construction work in hospitals: identification of risks and implementation of management precautions 2011. French Society for Medical Mycology website. https://sf2h.net/wp-content/uploads/2016/04/SF2H-SFMM_fungal-infections-guidelines-2011.pdf. Accessed May 23, 2021.

[10] Sehulster L, Chinn RY. Guidelines for environmental infection control in health-care facilities. Recommendations of CDC and the Healthcare Infection Control Practices Advisory Committee (HICPAC). Morbid Mortal Wkly Rept Recommend Rept 2003;52:1–42. 12836624

