# Supplementary material for: Surveillance practices and air-sampling strategies to address healthcare-associated invasive mold infections in Society for Healthcare Epidemiology of America (SHEA) Research Network hospitals—United States, 2020
Source: Infect Control Hosp Epidemiol. 2021 Jul 16;43(11):1708–11. doi: 10.1017/ice.2021.285 (PMC8761226; doi:10.1017/ice.2021.285)
Supplement: Supplementary file 1 [file S0899823X21002853sup001.docx]

| **Supplementary Table 1. Characteristics of Society for Healthcare Epidemiology of America (SHEA) Research Network Acute Care Hospitals (n = 37) — United States, 2020** | | |
| --- | --- | --- |
| **Characteristic** | **no.** | **%** |
| **Survey respondent role at institution** |  |  |
| Hospital epidemiologist | 27 | 73.0 |
| Infection prevention program manager | 5 | 13.5 |
| Infection preventionist | 2 | 5.4 |
| Infectious disease specialist | 2 | 5.4 |
| Senior leader (e.g., CMO, other C-suite) | 1 | 2.7 |
| **Hospital type** |  |  |
| Academic | 25 | 67.6 |
| Private, non-profit | 8 | 21.6 |
| Federal (e.g., VA, Department of Defense) | 2 | 5.4 |
| Local public governmental (e.g., county hospital) | 1 | 2.7 |
| Missing | 1 | 2.7 |
| **Number of beds** |  |  |
| ≤200 | 4 | 10.8 |
| 201–500 | 11 | 29.7 |
| >500 | 16 | 43.2 |
| Missing | 6 | 16.2 |
| **Programs and units available at hospital** |  |  |
| Medical, surgical, or other intensive care unit | 35 | 94.6 |
| Neonatal intensive care unit | 31 | 83.8 |
| Hematology/oncology unit | 30 | 81.1 |
| Solid organ transplant program | 29 | 78.4 |
| Bone marrow/stem cell transplant program | 24 | 64.9 |
| Burn program | 11 | 29.7 |
| None of the above | 0 | 0.0 |
| **Number of intensive care units** |  |  |
| 1–3 | 12 | 32.4 |
| 4–6 | 10 | 27.0 |
| 7–9 | 7 | 18.9 |
| ≥10 | 2 | 5.4 |
| Missing | 6 | 16.2 |

| **Supplementary Table 2. Collaboration among Stakeholders in Prevention and Mitigation of Healthcare-associated Invasive Mold Infections at Society for Healthcare Epidemiology of America (SHEA) Research Network Acute Care Hospitals (n = 37) — United States, 2020** | | | | | | | |
| --- | --- | --- | --- | --- | --- | --- | --- |
| **Characteristic** | **Total**  **(n = 37)** | | **Hospital type** | | | | ***P* value** |
|  |  |  | **Academic (n = 25)** | | **Non-Academic (n = 12)** | |  |
|  | **no.** | **%** | **no.** | **%** | **no.** | **%** |  |
| **Access to industrial hygienist consultation** |  |  |  |  |  |  | 0.70 |
| Industrial hygienist on staff | 10 | 27.0 | 8 | 32.0 | 2 | 16.7 |  |
| Ongoing contract with an external industrial hygienist | 7 | 18.9 | 4 | 16.0 | 3 | 25.0 |  |
| Do not have an industrial hygienist on staff or contracted, but can consult one as needed | 13 | 35.1 | 9 | 36.0 | 4 | 33.3 |  |
| Do not have access to an industrial hygienist | 1 | 2.7 | 1 | 4.0 | 0 | 0.0 |  |
| Unknown | 6 | 16.2 | 3 | 12.0 | 3 | 25.0 |  |
| **Collaboration between infection prevention team and staff who oversee hospital’s HVAC systems** |  |  |  | 3 |  |  | 0.33 |
| Not meeting regularly, but have met within the past year to address issues related to HVAC and potential exposure to airborne contaminants | 18 | 48.6 | 13 | 52.0 | 5 | 41.7 |  |
| Regularly scheduled recurring meeting(s) to address issues related to HVAC and potential exposure to airborne contaminants | 12 | 32.4 | 9 | 36.0 | 3 | 25.0 |  |
| Have not met within the past year to address issues related to HVAC and potential exposure to airborne contaminants | 1 | 2.7 | 0 | 0.0 | 1 | 8.3 |  |
| Unknown | 6 | 16.2 | 3 | 12.0 | 3 | 25.0 |  |
| **Presence of a project risk team that reviews proposed maintenance, renovation, and construction activities that pose an increased risk of generating or releasing microbial contamination** | 31 | 83.8 | 22 | 88.0 | 9 | 75.0 | 0.37 |
| **Presence of a system to prevent the placement of immunocompromised persons**^a^ **in negative pressure rooms** | 24 | 64.9 | 18 | 72.0 | 6 | 50.0 | 0.27 |
| Abbreviations: HVAC = heating, ventilation, and air conditioning; PCR = polymerase chain reaction | | | | | | | |
| ^a^ National Healthcare Safety Network (NHSN) defines immunocompromised patients as: • those with neutropenia defined as absolute neutrophil count or total white blood cell count (WBC) <500/mm^3^ • those with leukemia, lymphoma or who are HIV-positive with CD4 count <200 • those who have undergone splenectomy • those who have a history of solid organ or hematopoietic stem cell transplant • those on cytotoxic chemotherapy • those on enteral or parenteral administered steroids (excludes inhaled and topical steroids) daily for >2 weeks on the date of event | | | | | | |  |
